# A Comprehensive Evaluation of Mechanical, Thermal, and Antibacterial Properties of PLA/ZnO Nanoflower Biocomposite Filaments for 3D Printing Application

**DOI:** 10.3390/polym14030600

**Published:** 2022-02-02

**Authors:** Tongsai Jamnongkan, Ornthiwa Jaroensuk, Anchan Khankhuean, Apirat Laobuthee, Natee Srisawat, Autchara Pangon, Rattanaphol Mongkholrattanasit, Pongthipun Phuengphai, Amnuay Wattanakornsiri, Chih-Feng Huang

**Affiliations:** 1 Department of Fundamental Science and Physical Education, Faculty of Science at Sriracha, Kasetsart University, Chonburi 20230, Thailand; ornthiwa.j@live.ku.th; 2 Department of Materials Engineering, Faculty of Engineering, Kasetsart University, Bangkok 10900, Thailand; por.bigsmile@gmail.com (A.K.); fengapl@ku.ac.th (A.L.); 3 Department of Textile Engineering, Rajamangala University of Technology Thanyaburi, Pathumthani 12110, Thailand; natee.s@en.rmutt.ac.th; 4 Nano Functional Fiber Research Team, National Nanotechnology Center, National Science and Technology Development Agency, Pathumthani 12120, Thailand; autchara@nanotec.or.th; 5 Faculty of Industrial Textiles and Fashion Design, Rajamangala University of Technology Phra Nakhon, Bangkok 10110, Thailand; rattanaphol.m@rmutp.ac.th; 6 Department of Fundamental Science, Faculty of Science and Technology, Surindra Rajabhat University, Surin 32000, Thailand; pongthipun.ph@srru.ac.th; 7 Department of Agriculture and Environment, Faculty of Science and Technology, Surindra Rajabhat University, Surin 32000, Thailand; amnuaywattanakornsiri@hotmail.co.th; 8 Department of Chemical Engineering, i-Center for Advanced Science and Technology (iCAST), National Chung Hsing University, Taichung 40227, Taiwan

**Keywords:** 3D printing, biocomposites, poly(lactic acid), ZnO nanoflowers, antibacterial property

## Abstract

Functionalities of 3D printing filaments have gained much attention owing to their properties for various applications in the last few years. Innovative biocomposite 3D printing filaments based on polylactic acid (PLA) composited with ZnO nanoflowers at varying contents were successfully fabricated via a single-screw extrusion technique. The effects of the varying ZnO nanoflower contents on their chemical, thermal, mechanical, and antibacterial properties were investigated using Fourier transform infrared spectroscopy (FTIR), differential scanning calorimetry (DSC), and tensile testing, as well as qualitative and quantitative antibacterial tests, respectively. It was found that the ZnO nanoflowers did not express any chemical reactions with the PLA chains. The degrees of the crystallinity of the PLA/ZnO biocomposite filaments increased when compared with those of the neat PLA, and their properties slightly decreased when increasing the ZnO nanoflower contents. Additionally, the tensile strength of the PLA/ZnO biocomposite filaments gradually decreased when increasing the ZnO nanoflower contents. The antibacterial activity especially increased when increasing the ZnO nanoflower contents. Additionally, these 3D printing filaments performed better against Gram-positive (*S. aureus*) than Gram-negative (*E. coli*). This is probably due to the difference in the cell walls of the bacterial strains. The results indicated that these 3D printing filaments could be utilized for 3D printing and applied to medical fields.

## 1. Introduction

3D printing is a relatively new technology and has become popular in the last few years. This technology can process a wide variety of materials and produce fully functional components, which are fabricated in a bottom-up design without molding [1]. Additionally, it can be applied in many fields, including robotics, automobile components, firearms, medical science, space engineering, and so on. [2,3,4]. Filament types are important in fabricating 3D printing products, and commodity filaments have been produced from several materials, such as polylactic acid (PLA), polycaprolactone (PCL), polyurethane (PU), and acrylonitrile butadiene styrene (ABS). Recently, the biodegradable filaments for 3D printing have been of interest to researchers due to their vast variety of applications and advantages [5]; one advantage is that they contribute to a decrease in environmental pollution when compared with synthetic ones [6,7,8,9].

Presently, synthetic filaments for 3D printing are commonly fabricated from petroleum-based polymers, such as polystyrene (PS), polyetherimide (PEI), and polyethylene (PE) [10,11]. In addition, it was found that many additive fillers were loaded into melted polymers to fabricate 3D printing composite filaments, e.g., particles [12], cellulose nanofibers [13], nanomaterials [14], and fibers [15,16,17], in order to improve their mechanical and other properties. Among these fillers, nanoparticles are broadly studied, but few studies report their specialty functions, particularly, the effects of particles’ shapes on their composite properties, including an antibacterial property. Presently, biocomposite filaments (fabricated from biodegradable matrices reinforced with fillers) are gaining considerable interest as environmentally friendly materials and for their potential in specific applications. Previous studies have stated that biocomposite films prepared from PLA and metal-based nanoparticles, e.g., silver (Ag) [18], zinc oxide (ZnO) [19], and titanium dioxide (TiO_2_) [20], showed good thermal and antibacterial properties.

ZnO nanofibers, owing to their high surface-to-volume ratio, low density, interconnected porous structures, and low toxicity to humans, have gained renewed interest for their potential application in various fields [21,22,23,24,25]. Additionally, their nanoparticle shapes would affect their chemical properties. The flower-like shape of ZnO, called a ZnO nanoflower, is one of the most interesting shapes for characterization because it exhibits a large surface area property. A simple method for producing ZnO nanoflowers is the electrospinning method, which is able to produce at room temperature under ambient conditions. Additionally, their size, shape, and morphology can be defined by optimizing the conditions of the spinning processes [26].

Generally, the morphology or shape of fillers in biocomposites directly affects their properties, including chemical, thermal, mechanical, and antibacterial properties. To our best knowledge, there have been no studies about ZnO nanoflower-based biocomposites that include their properties. In this study, we presented a new paradigm in 3D printing technology with the fabrication of biocomposite 3D printing filaments based on PLA and ZnO nanoflowers in order to improve their antibacterial properties as a novel biomaterial for biomedical applications. We investigated the effects of varying ZnO nanoflower contents on their chemical, thermal, mechanical, and antibacterial properties using Fourier transform infrared spectroscopy (FTIR), differential scanning calorimetry (DSC), and tensile tests, as well as qualitative and quantitative antibacterial tests, respectively.

## 2. Materials and Methods

### 2.1. Materials

Commercial-grade poly(lactic acid) (PLA) was obtained from NatureWorks LLC (Minnetonka, MN, USA) with the trade name of Ingeo 3100 HP (*M*_w_ = 140,000 g/mol). Zinc acetate was purchased from LOBA Chemie Co., Ltd., Bombay, India. Other chemical agents were of analytical-grade purity and used as received.

### 2.2. Preparation of 3D Printing Filaments

ZnO nanoflowers were successfully fabricated by electrospinning from a suspension of zinc acetate used as a precursor via a calcination process [26]. Then, 3D printing filaments of PLA loaded with varying ZnO nanoflower contents were prepared by an extrusion method as follows. PLA pellets were firstly dried at 80 °C for 12 h and then milled into powder form. Then, the powder was mixed with ZnO nanoflowers and incorporated utilizing a high-speed mixer. Subsequently, the dry mixture was fed into the hopper of a lab-scale single-screw extruder (LTE 26-44, Labtech Engineering, Samut Prakan, Thailand) with a screw diameter of 26 mm and a length–diameter ratio of 44. During the extrusion process, the fed temperature at the first zone of the extruder and die temperature were consecutively set to 165 °C and 180 °C. Screw rotation speed was constantly maintained at 20.75 rpm to match the traction system for producing the 3D filaments. In this paper, the 3D printing filament samples are referred to as PLA, PLA/ZnO-1, PLA/ZnO-3, and PLA/ZnO-5, corresponding to the neat PLA and the PLA composited with ZnO nanoflowers at contents of 1, 3 and 5 wt%, respectively.

### 2.3. Fourier Transform Infrared Spectroscopy

FTIR was performed to examine the presence of a chemical reaction between ZnO nanoflower molecules and PLA molecular chains. All 3D printing filaments were dried at 80 °C for 12 h to remove the moisture from the ambient air, then were cut into small pieces for FTIR investigation. An FTIR spectrometer (FTIR-4100, Jasco, Tokyo, Japan) in ATR mode and equipped with a diamond crystal was used for the tests. All the spectra were recorded in transmittance mode with 4 cm^−1^ resolution in the wavenumber range of 4000 cm^−1^–400 cm^−1^ under ambient conditions.

### 2.4. Differential Scanning Calorimetry

The thermal properties of these 3D printing filaments were characterized using a DSC technique (model DSC 200 F3, Netzsch, Selb, Wunsiedel, Germany). All the samples were in a sealed aluminum pan, and each sample used was approximately 5 mg. They were sequentially heated from 30 to 200 °C and cooled to 25 °C, and then reheated to 200 °C at the rate of 10 °C/min under a nitrogen flow of 50 mL/min. After that, the degree of crystallinity of the 3D printing filaments ($\chi_{c}$) was calculated by Equation (1) [27].

(1) $$\chi_{c}/\%=\frac{\Delta H_{m}}{w\times \Delta H_{m}^{o}}\times 100$$

where, $∆H_{m}^{{}^{\circ}}$ is the melting enthalpy for 100% crystalline PLA (93 J/g) [28], $∆H_{m}$ is the melting enthalpy for the 3D printing filaments, and w is the mass fraction of PLA in the biocomposites. 

### 2.5. Tensile Testing

Tensile tests of all the 3D printing filaments were performed on a universal testing machine (model 5560, Instron, Norwood, MA, USA). A load cell of 25 kN was employed for testing all the samples. All the 3D printing filament specimens were fabricated into a rectangular shape (30 mm × 10 mm × 0.4 mm), according to the ASTM D638, via a 3D printer (model: RAISE3D N2). The conditions of fabrication were as follows: 0.1% of filled density with X-direction of printing, print speed of 40 mm/s, and printing and bed temperatures of 215 °C and 60 °C, respectively. Crosshead speed and gauge length were set at 5 mm/min and 25 mm, respectively. Five specimens of each sample were determined, and their results were averaged to obtain a mean value.

### 2.6. Antibacterial Testing

Antibacterial activities in both qualitative and quantitative tests were investigated in each 3D printing filament sample to evaluate their efficacies against Gram-negative (*E. coli*) and Gram-positive (*S. aureus*) bacterial strains, using the disk diffusion susceptibility and colony counting methods, respectively. The qualitative antibacterial activity of the samples was investigated as follows. The bacteria used were cultured in an LB medium and then incubated at 30 °C for 24 h. A 10^−2^ dilution of the incubated bacteria, approximately 10^8^ CFU/mL, was transferred to an LB agar plate. Disc-shapes of 5.00 mm diameter were cut from the samples. The 3D printing filament sample of neat PLA was used as a control for comparing the potential antibacterial properties with the other samples. Next, the cut-out discs were placed on the LB agar plates spread with bacteria and incubated for 24 h at 37 °C. After the incubation period, the diameters of the bacterial inhibition zone were measured in mm with a transparent ruler. These experiments were performed in triplicate.

In addition, the potential quantitative antibacterial activity of the samples was performed as follows. The bacterial cells, both *S. aureus* and *E. coli*, were grown overnight in an LB broth medium at 30 °C. Then, the 10^−2^ dilution suspensions of grown bacteria were prepared, and the given content of the samples at the ratio of 1:1 by weight was added to the suspensions. The 3D printing filament sample from the neat PLA was also used as a control. The suspensions were shaken in a rotary shaker (model XY-80, Taitec, Koshigaya, Saitama, Japan) at the constant speed of 120 rpm for 3 h at 30 °C and were diluted 10-fold repeatedly. A 100 μL aliquot of the bacterial cell suspension was spread on the LB agar nutrient plates using a glass stick. The plates were incubated at 37 °C for 24 h. Then, the number of viable bacterial cells on the plates was counted and then calculated to the initial number of bacterial cells before dilution. The antibacterial efficacy or the percent reduction of bacteria was also calculated by Equation (2):

(2) $$R=\frac{\left(A-B\right)}{A}\times 100\%$$

where *R* is the percent reduction in the number of viable bacteria, *A* is the number of bacterial cells in the presence of the 3D printing filaments from the neat PLA (CFU/mL), and *B* is the number of bacterial cells in the presence of those filaments from PLA/ZnO biocomposites (CFU/mL). All of the tests were also repeated in triplicate.

## 3. Results and Discussion

### 3.1. Feasible Preparation of 3D Printing Filaments

**Figure 1:**
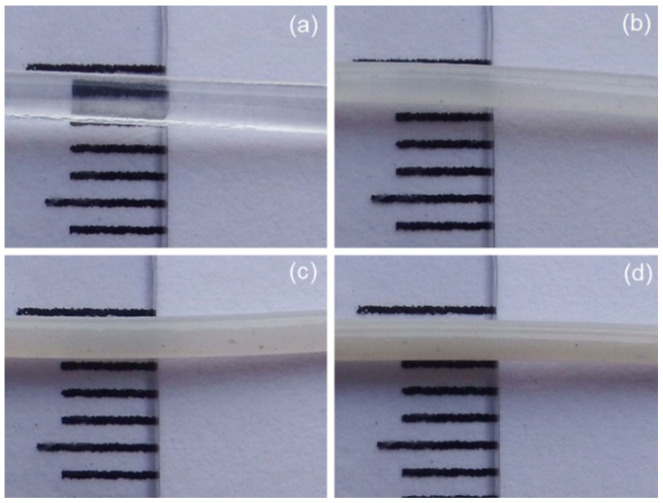
Optical images of 3D printing filaments from (**a**) neat PLA and PLA composited with ZnO nanoflowers at contents of 1 wt% (**b**), 3 wt% (**c**) and 5 wt% (**d**), respectively.

**Figure 2:**
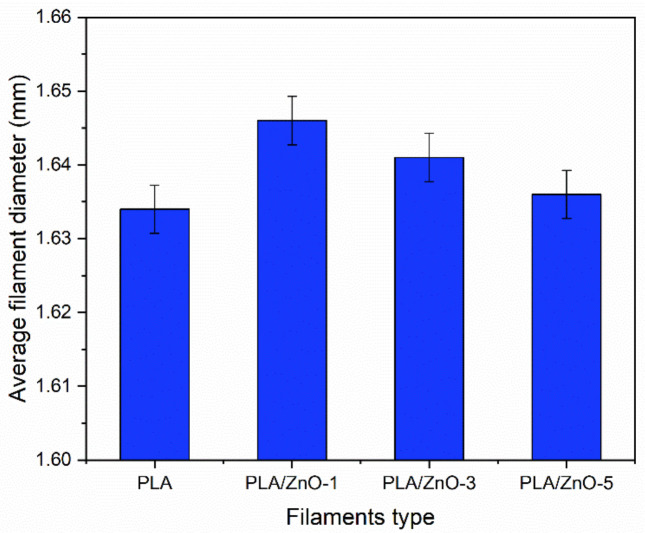
Average filament diameters from neat PLA and PLA composited with ZnO nanoflowers at varying contents.

The 3D printing filaments were successfully fabricated by a single-screw extrusion process, as depicted in Figure 1. From the optical images, the results indicate that the single-screw extrusion could produce the 3D printing filaments, with roughly uniform diameters from all four samples. The images of the four filaments also appear to be similar, but their color shades became more white-turbid with increasing ZnO nanoflowers, as shown in Figure 1. It is well known that the diameter of 3D printing filaments will affect a production process’s ability. Therefore, we measured their diameters and found that the average diameters of 3D printing filaments fabricated from PLA incorporated with ZnO nanoflowers exhibited slightly larger than those from the neat PLA filament. However, the diameters of the PLA/ZnO filaments decreased when increasing the ZnO nanoflower contents, as depicted in Figure 2. This is probably due to the distribution of nanoflowers within the PLA matrix. These results indicated that ZnO nanoflowers affected the mechanical and thermal properties. Therefore, it is worthwhile to further examine the effects of ZnO nanoflower contents on these properties.

### 3.2. Fourier Transform Infrared Spectroscopy

**Figure 3:**
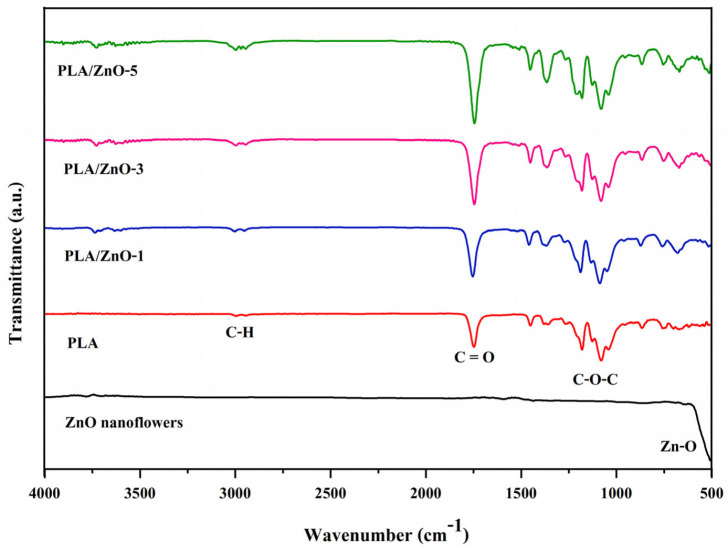
FTIR spectra of ZnO nanoflowers, neat PLA, and PLA/ZnO biocomposite filaments.

Compositions and chemical structures of all the 3D printing filaments were verified by FTIR. We compared the neat ZnO nanoflowers and the PLA/ZnO filaments, as illustrated in Figure 3. FTIR spectra of ZnO nanoflowers showed that a strong absorption peak appeared at approximately 544 cm^−1^, thereby signifying the presence of Zn-O bonding [29,30,31]. In the case of the neat PLA, the peak displayed at around 2921 cm^−1^, signifying the presence of CH stretching. Absorption peaks also appeared at around 1600, 1187 and 1072 cm^−1^, relating to the vibration of -C=O stretching and the ester -C-O-C- symmetric stretching and asymmetric stretching bands, respectively [32]. The FTIR spectra of 3D printing filaments from PLA composited with ZnO nanoflowers showed absorption characteristic peaks of both ZnO and PLA molecules with essentially no additional peaks. These results demonstrate that no chemical reaction occurred between ZnO nanoflowers and PLA chains. Additionally, the peak around 3300 cm^−1^ becomes stronger and broader with increasing ZnO nanoflowers, indicating the formation of increasing interactions of hydrogen bonds between ZnO nanoflowers and PLA chains.

### 3.3. Thermal Property Analysis

**Figure 4:**
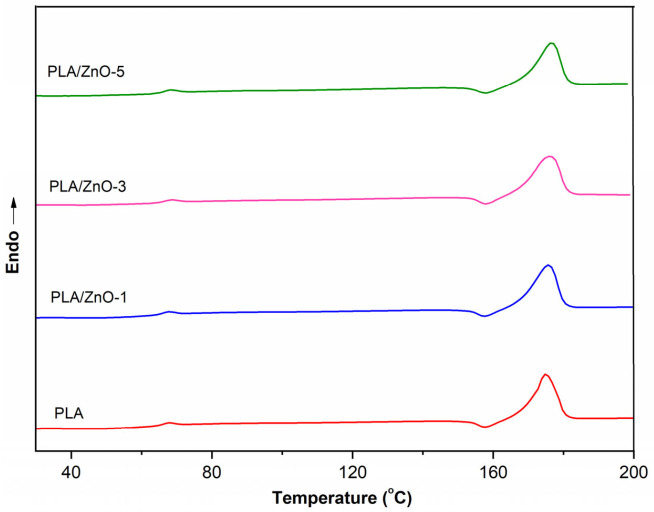
DSC thermograms of neat PLA and PLA/ZnO biocomposite filaments.

Differential scanning calorimetry (DSC) was performed in order to detect glass transitions and melting temperatures of all the PLA/ZnO biocomposite filaments. DSC thermograms of neat PLA and PLA composited with varying contents of ZnO nanoflowers are shown in Figure 4, and the results are collected in Table 1. The effect of ZnO nanoflowers on the thermal properties of the samples was characterized by the glass transition temperature (T_g_) and the melting temperature (T_m_) together with the degree of crystallinity (*χ_c_*). The DSC curves of 3D printing filaments displayed endothermic T_g_ regions and endothermic T_m_ peaks. This shows that the endothermic T_g_ peak is related to PLA chain relaxation [33]. Table 1 shows that the neat PLA filament has lower T_g_ and T_m_ values when compared with the filaments of PLA composited with ZnO nanoflowers. However, the content of ZnO nanoflowers within a PLA matrix slightly affected the thermal properties of the 3D printing filaments in both T_g_ and T_m_ values, as shown in Table 1. 

Moreover, the Tm value of PLA/ZnO-5 was shifted a bit towards higher temperatures compared with PLA/ZnO-3 and PLA/ZnO-1, respectively, as shown in Figure 4. This behavior was due to the distribution of ZnO nanoflowers in the PLA matrix, relating to the degree of crystallization via the nucleating efficiency process [34]. However, the ZnO nanoflowers content did not show a significant influence on the thermal properties of the filaments of PLA composited with ZnO nanoflowers. There were no significant changes in the thermal properties of the PLA/ZnO biocomposite filaments. This means that ZnO nanoflowers have little influence on the intermolecular interactions or chain flexibility of PLA polymer chains, in agreement with the result of Müller and his colleagues [35]. However, we found that the degrees of the crystallinity of the PLA/ZnO biocomposite filaments increased when compared with those of the neat PLA. Additionally, they slightly decreased when increasing the ZnO nanoflower contents. This means that the PLA/ZnO biocomposite filaments became amorphous and were more liable to undergo higher ZnO nanoflower contents, which might be from the dispersibility of nanofillers within the matrix of PLA.

### 3.4. Tensile Testing

**Figure 5:**
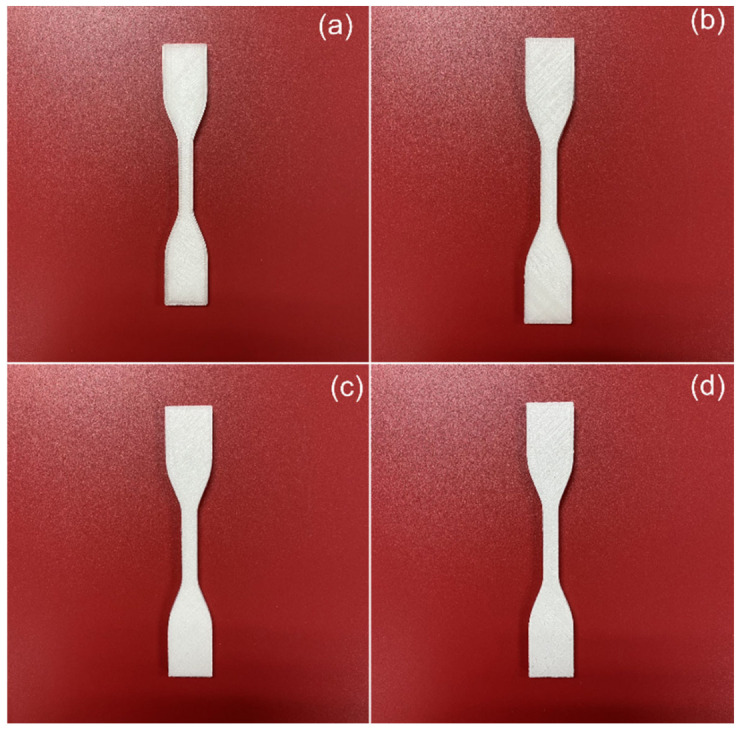
Optical images of ASTM D638-14 type IV tensile test specimens of (**a**) neat PLA, (**b**) PLA/ZnO-1, (**c**) PLA/ZnO-3, and (**d**) PLA/ZnO-5, respectively.

**Figure 6:**
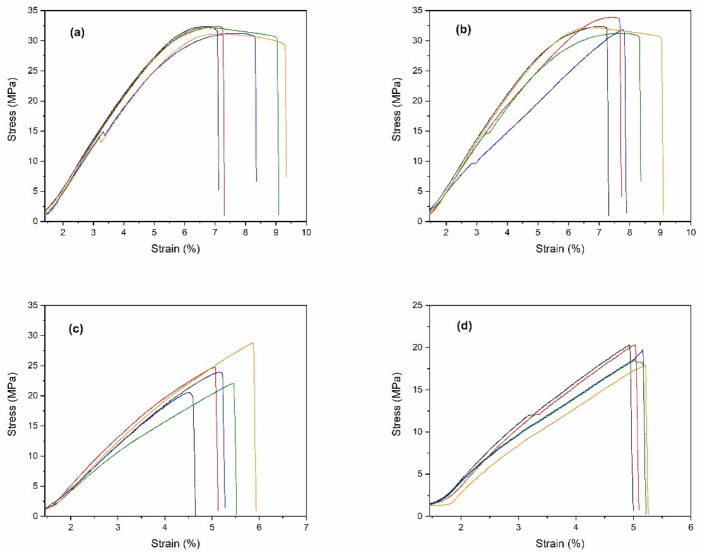
Stress-strain curves of: (**a**) neat PLA, (**b**) PLA/ZnO-1, (**c**) PLA/ZnO-3, and (**d**) PLA/ZnO-5, respectively. Each curve indicates the sample number.

**Figure 7:**
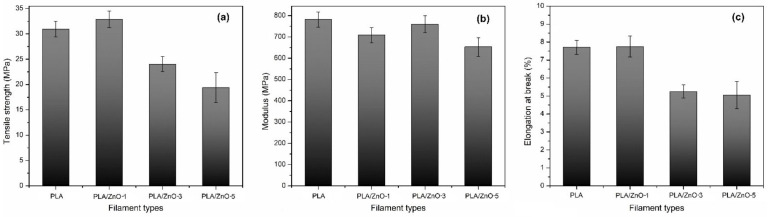
Effect of varying ZnO nanoflower contents on the mechanical properties: tensile strength (**a**), modulus strength (**b**), and elongation at break (**c**), of PLA and PLA/ZnO biocomposite filaments.

All the 3D printing filaments were fabricated into specimens for tensile testing, according to the ASTM D638-14 type IV [36], as shown in Figure 5. The stress-strain curves for all specimens were obtained and are shown in Figure 6. From these curves, the tensile strength, modulus, and elongation at break were obtained. The mechanical properties of the PLA/ZnO biocomposite filaments are presented in Figure 7 and Table 2. The tensile strength of composite materials generally relies on the stress transfer capacity between fillers and matrices, which are significantly affected by their interfacial adhesion [37,38]. We found that the tensile strength of the PLA/ZnO biocomposite filaments gradually decreased with increasing ZnO nanoflower contents (Figure 6a). The PLA/ZnO-5 encountered a decrease in tensile strength because of the higher ZnO nanoflower contents, caused by filler agglomeration during the melt extrusion process, resulting in poor interfacial adhesion of particles and matrix [39,40]. From Table 2, the modulus strength of the PLA/ZnO biocomposite filaments decreased when the ZnO nanoflower contents increased. We found that PLA/ZnO-1 exhibited the highest elongation at break among the four specimens (53.46% higher than that of PLA/ZnO-5 (Figure 6c)). This result is in agreement with the result of the thermal properties, relating to the degree of distribution of nanodomain within polymer matrices [41]. 

### 3.5. Antibacterial Testing

**Figure 8:**
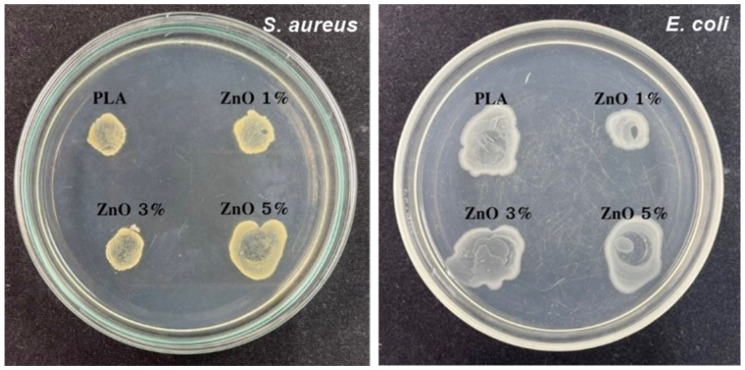
Inhibition zones of antibacterial activities of neat PLA and PLA/ZnO biocomposite filaments.

As a potential application, the neat PLA and PLA/ZnO biocomposite filaments were tested as a bactericidal action. Antibacterial activities were performed by agar well-diffusion and colony unit counting methods against Gram-positive (*S. aureus*) and Gram-negative (*E. coli*) pathogenic bacteria, as depicted in Figure 8, and the detailed results of the antibacterial efficacies are shown in Table 3. From the experimental results, the sizes of the inhibition zones were varied with respect to the type of bacterial strain. This is probably due to the differences in the bacterial surface characteristics. It is well known that Gram-positive and Gram-negative bacteria are different when observing their cell walls. Gram-positive bacteria have a thick, multi-layered peptidoglycan (negatively charged), no lipopolysaccharide, and the presence of teichoic acid. In contrast, Gram-negative bacteria have a thin, single-layered peptidoglycan, containing high lipopolysaccharides and an absence of teichoic acid. All these different cell surface compositions result in explicit differences in the sensitivity of bacteria to ZnO nanoflowers [42,43,44].

**Figure 9:**
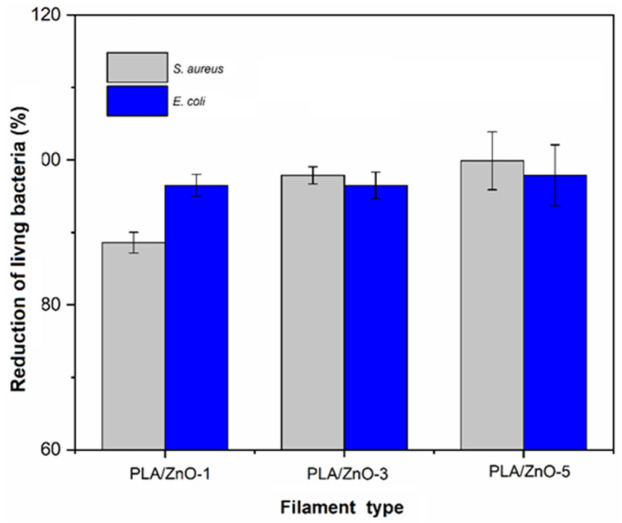
Percent antimicrobial efficacies for *S. aureus* and *E. coli* of PVA/ZnO composite filaments.

More precisely, the results showed that the percent antibacterial efficacies that the colony unit counting method depicted depended on the ZnO nanoflower contents within the PLA matrix. The percent antibacterial efficacies enhanced when increasing the ZnO nanoflower contents in both Gram-positive and Gram-negative bacteria, as shown in Figure 9. Additionally, it was observed that the numbers of viable bacteria cells of *S. aureus* exhibited lower than those of *E. coli* at the given ZnO nanoflower contents. These findings are in good agreement with the reported studies [45,46,47]. It is well known that the antibacterial activity of ZnO nanoflowers is dependent on their contents, and that a higher content of ZnO nanoflowers shows higher bactericidal activity owing to their higher surface-to-volume ratios for reacting with bacterium cells [42,48,49].

## 4. Conclusions

The antibacterial functionality of PLA-composited ZnO nanoflowers was assessed at the various concentrations, and 3D printing filaments were successfully fabricated via a single-screw extrusion technique. We found that the contents of ZnO nanoflowers incorporated with the PLA matrix did not significantly affect the thermal and surface-morphological properties. The T_g_ and T_m_ values of the 3D printing filaments from PLA composited with ZnO nanoflowers shifted a bit towards higher temperatures when compared with those from the neat PLA. However, they encountered a decrease in tensile strength because of higher ZnO nanoflower contents, caused by filler agglomeration during the melted extrusion process, which then resulted in poor interfacial adhesion of ZnO nanoflowers and the PLA matrix. Investigating an antibacterial property of 3D printing filaments is of the utmost importance. We found that the antibacterial efficacy directly depended on the ZnO nanoflower contents. The antibacterial activities increased with increasing ZnO nanoflower contents, and these 3D printing filaments were better against Gram-positive than Gram-negative bacteria because of differences in their cell walls. We tentatively suggest that our results indicate the possibility that the ZnO nanoflower could be released from the PLA filaments. Further investigations into this possibility are currently underway. Additionally, the functional 3D print with the proposed composition will be studied, and we intend to address this phenomenon in detail in a future publication. Finally, FTIR spectra indicated that the ZnO nanoflowers were merely distributed in the PLA matrix without any chemical bonding of ZnO molecules with PLA chains, but entirely dispersed in the PLA matrix. This study showed that these biocomposite 3D printing filaments based on PLA and ZnO nanoflowers can be applied in biomedical fields. 

## Figures and Tables

**Table 1:** Thermal properties of 3D printing filaments from neat PLA and PLA composited with ZnO nanoflowers at varying contents.

Samples	T_g_ (°C)	T_m_ (°C)	*χ_c_* (%)
PLA	66.80	175.60	66.22
PLA/ZnO-1	66.60	176.00	69.66
PLA/ZnO-3	66.90	176.50	67.15
PLA/ZnO-5	66.70	176.80	66.61

**Table 2:** Mechanical properties of 3D printing filaments from neat PLA and PLA composited with ZnO nanoflowers at varying contents.

Samples	Tensile Strength (MPa)	Modulus Strength (MPa)	Elongation at Breaks (%)
PLA	30.95	782.25	7.71
PLA/ZnO-1	32.85	708.50	7.75
PLA/ZnO-3	24.02	760.00	5.25
PLA/ZnO-5	19.40	653.00	5.05

**Table 3:** Antibacterial efficacies of PLA/ZnO composite filaments.

Samples	*S. aureus*		*E. coli*	
	Number of Living Bacteria (CFU/mL)	Reduction of Living Bacteria (%)	Number of Living Bacteria (CFU/mL)	Reduction of Living Bacteria (%)
PLA	1.29 × 10^8^	0.0	1.39 × 10^8^	0.0
PLA/ZnO-1	1.47 × 10^7^	88.6	4.80 × 10^6^	96.5
PLA/ZnO-3	2.26 × 10^6^	97.9	4.90 × 10^6^	96.5
PLA/ZnO-5	9.70 × 10^3^	99.9	2.85 × 10^6^	97.9

## Data Availability

Not applicable.
